# Robotic Surgical Outcomes in Endometrial Cancer: Does Class III Obesity Matter?

**DOI:** 10.3390/cancers18040706

**Published:** 2026-02-22

**Authors:** Vito Andrea Capozzi, Asya Gallinelli, Elisa Scarpelli, Stefano Restaino, Giuseppe Vizzielli, Roberto Berretta

**Affiliations:** 1 Department of Obstetrics and Gynecology, University of Parma, 43125 Parma, Italy; 2 Department of Medical and Surgical Sciences (DIMEC), University of Bologna, 40126 Bologna, Italy; 3 Department of Maternal and Child Health, “Santa Maria della Misericordia” University Hospital, Azienda Sanitaria Universitaria Friuli Centrale (ASUFC), 33100 Udine, Italy

**Keywords:** endometrial cancer, obesity, class III obesity, robotic surgery, minimally invasive surgery, sentinel lymph node mapping, perioperative outcomes

## Abstract

Background/Objective: Women with Class III obesity (BMI ≥ 40 kg/m^2^) have a lifetime risk of endometrial cancer (EC) as high as 10–15%. However, evidence focused specifically on Class III obese patients remains limited. This study evaluated the surgical feasibility and safety of robotic surgery in Class III obese women with EC. Methods: A single-center retrospective study was conducted at the ESGO-accredited University Hospital of Parma (Italy) from October 2021 to February 2025. All women had apparent early-stage EC and underwent robotic hysterectomy with bilateral salpingo-oophorectomy and sentinel lymph node (SLN) mapping with Indocyanine Green. Patients were categorized into three BMI groups: Group A (BMI ≥ 40 kg/m^2^), Group B (BMI 30–39 kg/m^2^), and Group C (BMI < 30 kg/m^2^). Perioperative variables—including operative time, estimated blood loss, conversion to laparotomy, intra- and postoperative complications, hospital and Intensive Care Unit (ICU) stay, and SLN mapping failure—were compared across groups. Results: A total of 109 women were included: 26 (23.9%) in Group A, 45 (41.3%) in Group B, and 38 (34.9%) in Group C. Class III obesity was not associated with higher intraoperative (*p* = 0.390) or postoperative (*p* = 0.805) complication rates. Conversion to laparotomy (*p* = 0.720), estimated blood loss (*p* = 0.123), ICU stay (*p* = 0.156), and hospital stay (*p* = 0.491) were superimposable across groups. Operative time was significantly longer in Group A (*p* = 0.003) compared to the other groups. Successful bilateral SLN mapping differed significantly across groups (*p* = 0.026), being lower in Group A (73.1%) compared to Group B (95.6%) and Group C (81.6%). Conclusions: Robotic surgery is safe and feasible in Class III obese EC patients, with perioperative morbidity comparable to that of lower BMI groups. Nevertheless, longer operative times and a lower rate of successful bilateral SLN mapping highlight the need for tailored strategies and further research to optimize nodal staging in severely obese women.

## Simple Summary

Obesity is a major risk factor for endometrial cancer (EC). Severe (Class III) obesity—defined as a body mass index (BMI) of 40 kg/m^2^ or higher—increases both the chance of developing EC and makes surgery more challenging. Robotic surgery is a minimally invasive technique that may help overcome these challenges, but data in patients with Class III obesity remain limited. In this study, we analyzed 109 patients with early-stage EC who underwent robotic hysterectomy with bilateral salpingo-oophorectomy and sentinel lymph node staging, and compared perioperative outcomes across the BMI groups (Group A (≥40 kg/m^2^), Group B (30–39 kg/m^2^), and Group C (<30 kg/m^2^)). We found that robotic surgery was surgically safe and feasible even in Class III obese patients, with no increase in intraoperative or postoperative complications compared to normal weight patients. However, Class III obese patients experienced longer operative times and lower rates of bilateral sentinel lymph node mapping compared to the other groups.

## 1. Introduction

The World Health Organization (WHO) recognizes obesity as a chronic, progressive, and relapsing disease and a global epidemic, with a worldwide prevalence of 20.8% in women [1,2]. In 2000, the WHO classified Obesity into Class I (BMI 30.0 to 34.9 kg/m^2^), Class II (BMI 35.0 to 39.9 kg/m^2^), and Class III (BMI ≥ 40 kg/m^2^) [3,4].

Furthermore, obesity is an independent risk factor for type 2 diabetes, cardiovascular disease, and gynecological cancers, especially endometrial cancer (EC) [1,5].

EC is the sixth most common cancer in women worldwide, with 417,000 new cases diagnosed per year, and the lifetime risk is around 3% [5]. However, its incidence is rising, especially in high-income countries [6,7], reflecting the increasing prevalence of obesity and the aging population [6,7]. Women with Class III obesity (BMI ≥ 40 kg/m^2^) have a lifetime risk of EC as high as 10–15%, which is comparable to the lifetime risk of lung cancer in smokers [8,9]. Lu et al. reported that EC risk increased in a dose-dependent manner with rising body mass index (BMI): for every 5 kg/m^2^ increase in BMI, the relative risk of EC rises by 50–54% [6,8]. Moreover, obesity has been reported as an independent contributor to poorer mortality outcomes among affected women [10].

Class III obese patients with EC face increased anesthesia risks, a higher burden of comorbidities, patient positioning difficulties, reduced visualization, and greater technical complexity [11,12,13]. The perioperative anesthetic risks highlight the need for surgical approaches that reduce physiological stress, such as minimally invasive and robotic techniques [14]. Minimally invasive surgery (MIS), including both laparoscopic and robotic approaches, is associated with reduced morbidity compared to laparotomy in obese women with EC [15].

However, conventional laparoscopy may encounter significant limitations in severely obese patients [16]. The increased thickness of the abdominal panniculus can lead to greater surgeon fatigue and restricted instrument mobility [17]. Moreover, higher pneumoperitoneum pressures are often required to maintain adequate exposure, potentially complicating ventilatory management and increasing perioperative anesthetic challenges [18].

Robotic-assisted surgery may help overcome several of these limitations. The platform allows for improved instrument dexterity, enhanced visualization, and better navigation around anatomical barriers [19]. In addition, the possibility of working at lower pneumoperitoneum pressures, combined with optimized Trendelenburg positioning, may facilitate ventilation and surgical exposure [19]. These technical advantages can expand surgical eligibility in patients who might otherwise be considered suboptimal candidates for laparoscopy, potentially reducing understaging. Indeed, robotic surgery has been associated with higher rates of successful sentinel lymph node mapping [20].

Despite these advances, evidence focusing specifically on Class III obese patients is scarce.

Most existing studies are retrospective, include small Class III sample sizes, and have heterogeneous outcome definitions [21,22,23]. These aspects reduce the generalizability of their findings. Standardized surgical protocols, clear BMI stratification, and structured follow-up address key limitations of previous research.

The present study, conducted at the European Society of Gynecologic Oncology (ESGO)-accredited University Hospital of Parma, specifically evaluates the surgical feasibility and safety of robotic surgery in Class III EC obese patients.

## 2. Materials and Methods

### 2.1. Study Design

A retrospective observational analysis was conducted on patients who underwent robotic surgical treatment for endometrial cancer at the ESGO-accredited University Hospital of Parma (Italy). The study period spanned from October 2021 to February 2025. The STROBE guidelines [24] were followed for this single-center retrospective observational investigation. The study was approved by the Ethics Committee of the University Hospital of Parma (approval code: 171/225/OSS/AOUPR).

### 2.2. Participants

All women with endometrial cancer treated surgically with robotic surgery were included. All cases were classified as FIGO apparent early-stage endometrial cancer [25] and underwent preoperative workup in accordance with ESGO guidelines [26]. Patients were initially categorized according to the World Health Organization (WHO) obesity classification: Class I (BMI 30–34.9 kg/m^2^), Class II (BMI 35–39.9 kg/m^2^), and Class III (BMI ≥ 40 kg/m^2^).

For analytical purposes, patients were subsequently stratified into three groups:

Group A: Class III obesity (BMI ≥ 40 kg/m^2^)

Group B: Class I–II obesity (BMI 30–39.9 kg/m^2^)

Group C: Non-obese women (BMI < 30 kg/m^2^)

This grouping strategy was adopted to isolate patients with extreme obesity (Class III).

Medical history, comorbidities, and biometric data were collected for all participants. Women younger than 18 years of age, those who did not provide consent for the use of their data for research purposes, and those with incomplete clinical, pathological, or surgical records were excluded.

### 2.3. Variables

The primary objective of the study was to assess the surgical feasibility of Class III obese EC patients (BMI ≥ 40 kg/m^2^) undergoing robotic surgery. The secondary objectives were to compare intraoperative and early postoperative complications (within 3 months) between Groups A, B, and C.

### 2.4. Data Sources/Measurement

BMI classification was based on the World Health Organization criteria, as described above. Patients were analyzed according to the predefined study groups (Group A: BMI ≥ 40 kg/m^2^; Group B: BMI 30–39.9 kg/m^2^; Group C: BMI < 30 kg/m^2^).

All included patients underwent total hysterectomy, bilateral salpingo-oophorectomy, and sentinel lymph node (SLN) staging for endometrial cancer, with indocyanine green used for injection. In cases of failed SLN mapping, the Sloan Kettering Cancer Center algorithm [27] was applied. In cases of failed SLN mapping and poor visibility of the surgical field in extremely obese cases, nodal staging was abandoned. In these cases, surgery was considered feasible, but patients were classified as having nodal staging ‘not attempted’.

During surgery, operative times and any complications such as bleeding, bladder, ureteral, intestinal, or nerve injuries were recorded. The Clavien-Dindo classification [28] was used for postoperative complications. A minimum follow-up period of 3 months was required, and patients underwent a gynecological follow-up examination 30 and 90 days after surgery.

### 2.5. Bias

In the absence of a univocal approved definition of surgical feasibility [29], we considered surgery to be feasible when the entire procedure of hysterectomy and bilateral salpingo-oopharectomy was performed using robotic surgery without conversion to either laparoscopy or laparotomy. Nodal staging was not included in the feasibility definition. In cases of failed SLN mapping combined with inadequate retroperitoneal visualization compromising surgical safety, nodal staging was not pursued. To address this bias, under-staged patients were recorded separately to distinguish procedural completeness from hysterectomy feasibility.

Since there is no standardized algorithm for early postoperative complications diagnosis, both a gynecological examination and pelvic ultrasound were performed 30 and 90 days after surgery. Second-level instrumental investigations (Computed Tomography and/or magnetic resonance) were performed only for uncertain cases or related to the patient’s symptoms.

### 2.6. Study Size

Considering a conversion rate of 19.0% in obese Class III patients undergoing robotic surgery vs. a 2.0% conversion rate in non-obese patients [30,31], an alpha error of 0.05, a beta error of 0.2, and a power of 80%, a sample size of at least 100 patients was required to reach statistical significance.

### 2.7. Setting

All cases were treated at the University Hospital of Parma, Italy. The hospital is an ESGO-accredited cancer center specializing in endometrial and ovarian cancers, as well as a referral center for gynecological oncology in the Emilia-Romagna region.

### 2.8. Statistical Analysis and Quantitative Variables

Continuous variables were reported as mean and standard deviation (SD) or, in the case of non-normal distribution, as median and interquartile range. Categorical variables will be reported as absolute frequencies and percentages. A one-way ANOVA test was used to analyze continuous variables within three groups. In case of a significant ANOVA, the Tukey post hoc test was used to confirm significance. The homogeneity of variance test was used to check whether the variances of multiple groups are equal. Categorical variables were analyzed through the Chi-square test. For significant variables (*p* < 0.05), Odds Ratio (OR) and 95% Confidence Interval (95% CI) were extrapolated. IBM SPSS Statistics for Windows, version 29.0.2.0 (IBM Corp., Armonk, NY, USA) was used for statistical analysis (20).

## 3. Results

**Figure 1:**
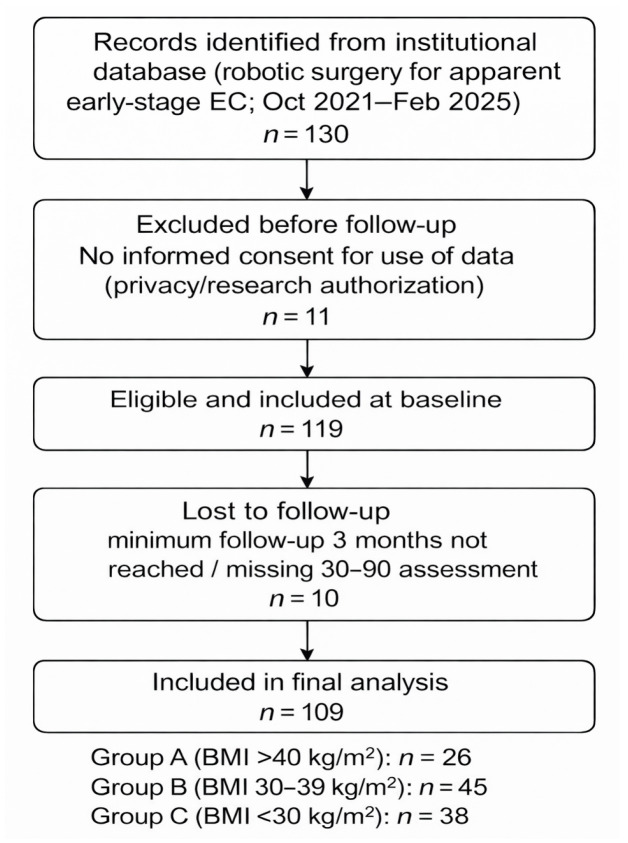
STROBE [25] flow chart of patient selection.

The participant flow is reported in the STROBE flowchart (Figure 1).

A total of 109 patients were included in the final analysis: 26 (23.9%) in Group A, 45 (41.3%) in Group B, and 38 (34.9%) in Group C. Mean age was 63.3 (±11.0) years, and mean BMI was 33.6 (±8.4). Baseline characteristics of the patients are reported in Table 1 and are not significantly different within the study groups.

No patient required laparoscopic conversion. Two patients (1.8% of the entire cohort) underwent laparotomy conversion and belonged to Groups B and C. The mean estimated blood loss (101.8 mL), mean day spent in postoperative Intensive Care Unit (ICU) (0.28 days), and mean hospital stay (3.57 days) were not different across the three groups (*p* = 0.123, *p* = 0.156, and *p* = 0.491, respectively). One intraoperative complication occurred during surgery in Group C (*p* = 0.39). A bowel perforation was found during lysis of adhesions due to previous surgery in a normal-weight patient. A total of 17 (15.6%) postoperative complications occurred: 5 (19.2%) in Group A, 7 (15.6%) in Group B, and 5 (13.2%) in Group C (*p* = 0.805). Complications are summarized in Table 2.

The mean operating time was different in the three groups (168.5 min vs. 143.2 min vs. 127.4 min, in Groups A, B, and C, respectively, ANOVA *p* = 0.004, 95% CI 0.011–0.205). The Tukey post hoc test showed that the significant difference in mean operative times was between Group A and C (*p* = 0.003). While the mean operating time between Group A and B did not differ (*p* = 0.08). No differences in mean operative times were observed between Groups B and C (*p* = 0.290).

**Figure 2:**
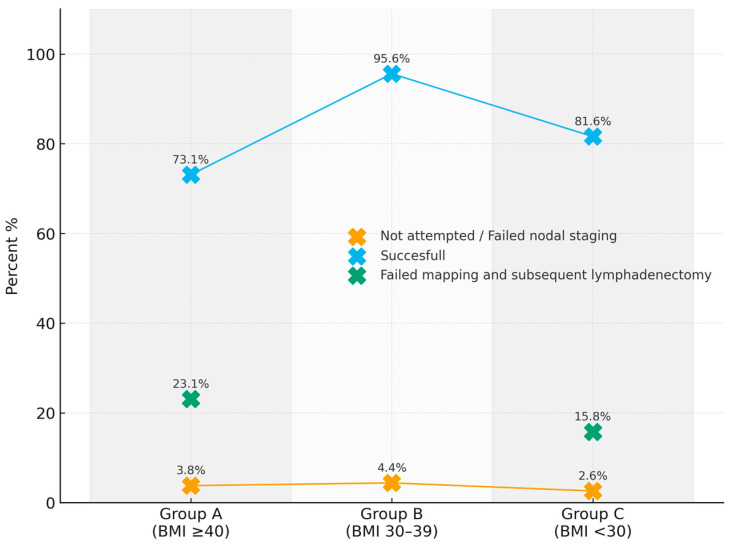
Distribution of sentinel lymph node (SLN) mapping outcomes according to body mass index (BMI) groups.

Nodal staging and SLN mapping are shown in Table 3 and Figure 2.

Bilateral SLN mapping was achieved in 93 patients (85.3%). However, successful bilateral SLN mapping was statistically lower in Group A (73.1%) with respect to Group B (95.6%) and Group C (81.6%), *p* = 0.026. Failed SLN mapping followed by side-specific lymphadenectomy occurred in 12 cases (11.0%). The rate differed significantly across groups (*p* = 0.006), with 6 patients (23.1%) in Group A, none in Group B, and 6 patients (15.8%) in Group C. Patients in Group A had 3.85 times the risk of failed SLN mapping (unadjusted OR 3.85, 95% CI 1.12–13.22 *p* = 0.002) compared to other groups. Four (3.7%) women had nodal staging not attempted: 1 (3.8%) patient in Group A, 2 (4.4%) in Group B, and 1 (2.6%) patient in Group C (*p* = 0.907).

## 4. Discussion

In this study, we evaluated the impact of obesity class on surgical feasibility and perioperative outcomes in a cohort of women undergoing robotic surgical treatment for endometrial cancer. Baseline characteristics were comparable across study groups, indicating that the observed differences are unlikely to be explained by measured confounders.

The study showed that Class III obesity significantly influenced specific intraoperative parameters. In particular, operative time increased progressively with higher BMI, with a statistically significant difference between Group A and Group C. Specifically, Class III obese patients had operating times approximately 40 min longer than normal weight patients. This observation aligns with existing evidence suggesting that increased adiposity may complicate surgical exposure and technical maneuvers. In line with our results, Eddib et al. reported a mean robotic operative time of 44 min longer in patients with a BMI > 35 compared to those with a BMI < 35 kg/m^2^ (266 vs. 222 min, respectively, *p* < 0.005) [32]. Additionally, Geppert et al. confirmed that patients with a BMI > 35 kg/m^2^ had a longer robotic operative time compared to open surgery [33]; however, this difference in operating time was nullified in the hands of experienced surgeons. Notably, all the perioperative outcomes analyzed—including estimated blood loss, postoperative ICU stay, overall length of hospitalization, and intra- and postoperative surgical complications—did not differ significantly across groups. The incidence of intraoperative complications was extremely low, with only one event recorded in Group C. Although no conversions were observed in Class III obese patients (Group A), the very low number of conversion events in the overall cohort limits the statistical power of this comparison and precludes definitive conclusions regarding differences between BMI groups. Similarly, the overall rate of postoperative complications did not differ significantly among the BMI categories. These data indicate that higher BMI was not associated with increased postoperative morbidity in this study. This supports the hypothesis that, despite adding technical challenges, obesity does not appear to compromise the overall safety of the procedure when managed by skilled surgical teams. While operative time was prolonged in higher BMI patients, this did not translate into worse short-term outcomes. Nevertheless, prolonged operative duration may have implications in terms of anesthetic management and should therefore be carefully addressed during preoperative counseling and surgical planning [34].

Nowadays, increasing evidence is emerging regarding the surgical safety of robotic surgery in morbidly obese patients in oncological fields. Recently, Arcieri et al. [35], in a retrospective cohort study, showed a comparable complication rate between obese patients treated with robotic surgery (mean BMI of 35.5 kg/m^2^) compared to non-obese patients (mean BMI of 24.0 kg/m^2^) treated with classic laparoscopy for EC or endometrial atypical hyperplasia. Furthermore, Papageorgiou et al. [36] confirmed our results with no intra- and postoperative complications reported, also in Class III obese EC patients who underwent robotic surgery. However, it should be emphasized that this low complication rate must be attributed to oncology referral centers with extremely experienced surgeons and staff.

One of the most relevant findings of this study concerns the impact of BMI on bilateral SLN mapping. We observed a significantly lower bilateral mapping success rate in Group A compared with Groups B and C, with patients in the highest BMI category exhibiting nearly a fourfold increased risk of mapping failure. This suggests that obesity may interfere with tracer diffusion, consistent with previous reports [37]. The increased need for side-specific lymphadenectomy in patients with higher BMI underscores the clinical implications of this limitation. In our high-volume center, which reports bilateral detection rates of approximately 90% and applies standardized SLN protocols, including reinjection in cases of initial mapping failure [38], the reduced mapping success observed in Class III obese patients is unlikely to be attributable to technical variability. Nevertheless, technical factors, such as tracer dose or injection technique, and patient-related anatomical factors, particularly increased adiposity and reduced retroperitoneal exposure, likely coexist and collectively influence mapping outcomes.

Finally, Group A had an equal number of nodal stagings not attempted due to inadequate surgical field compared to the other groups. Only one Class III obese patient did not undergo nodal staging due to a suboptimal view of the surgical field and belonged to the low-risk group according to the ESGO molecular risk-group classification [26]. This aspect is of extreme importance because, although Group A had a lower rate of bilateral SLN mapping, women did not show a higher rate of nodal surgical understaging than Groups B and C, with consequent impact on adjuvant therapy.

The ongoing RObese trial (NCT05974995) [30] is evaluating robotic versus conventional laparoscopic surgery in patients with endometrial cancer. The 2029 results may further clarify the role of robotic surgery, particularly in obese patients, and provide additional insights into nodal staging strategies in this population.

The present study benefits from several strengths, including a well-characterized cohort, homogeneous baseline features, and the inclusion of only patients with endometrial cancer. In addition, a large number of Class III obese patients were included. However, some limitations must be considered. The unmeasured factors—such as body fat distribution or functional patients’ status—may have influenced the results. The single-center and the retrospective design may also limit the generalizability of the findings.

## 5. Conclusions

In conclusion, Class III obese EC patients showed increased robotic operative time and reduced SLN bilateral detection rate, without a corresponding rise in perioperative complications compared to normal weight and obese Class I and II patients. Moreover, unattempted nodal staging did not appear to differ across BMI groups in this cohort, although this finding should be interpreted cautiously given the limited number of events. These findings reinforce the relevance of BMI as a preoperative factor in surgical planning and patient counseling, and suggest that robotic surgery in Class III obese patients can be performed safely in experienced centers. Future studies with larger, multicenter cohorts are needed to validate these results and to identify strategies that could optimize SLN detection in obese patients.

## Figures and Tables

**Table 1:** Baseline Characteristics.

Variable	Total (*n* = 109)	Group A (BMI ≥ 40) *n* = 26	Group B (BMI 30–39.9) *n* = 45	Group C (BMI < 30) *n* = 38)	*p*-Value
Age, mean ± SD (years)	63.3 ± 11.0	60.5 ± 11.8	66.4 ± 9.8	61.7 ± 11.1	0.062
CA125 positive, *n* (%)	11 (10.1)	3 (11.5)	5 (11.1)	3 (7.9)	0.855
Grading, *n* (%)					0.471
G1	44 (40.4)	11 (42.3)	18 (40.0)	15 (39.5)	
G2	48 (44.0)	13 (50.0)	21 (46.7)	14 (36.8)	
G3	17 (15.6)	2 (7.7)	6 (13.3)	9 (23.7)	
Final pathological stage (FIGO 2023), *n* (%)					0.329
Stage I	78 (71.6)	21 (80.8)	32 (71.1)	25 (65.8)	
Stage II	15 (13.8)	3 (11.5)	4 (8.9)	8 (21.1)	
Stage III	16 (14.7)	2 (7.7)	9 (20.0)	5 (13.2)	
Stage IV	0 (0)	0 (0)	0 (0)	0 (0)	

SD: Standard deviation. BMI: Body mass index.

**Table 2:** Patient complications.

Variable	Total (*n* = 109)	Group A (BMI ≥ 40, *n* = 26)	Group B (BMI 30–39.9, *n* = 45)	Group C (BMI < 30, *n* = 38)	*p*-Value
Conversion to laparotomy, *n* (%)	2 (1.8)	0 (0)	1 (2.2)	1 (2.6)	0.720
Estimated blood loss, mean ± SD (mL)	101.8 ± 148.7	153.8 ± 256.9	84.4 ± 92.2	86.8 ± 86.7	0.123
Operative time, mean ± SD (min)	143.7 ± 49.5	168.5 ± 54.7	143.2 ± 40.5	127.4 ± 49.7	0.004
ICU stay, mean ± SD (days)	0.28 ± 1.15	0.62 ± 0.70	0.29 ± 1.66	0.05 ± 0.32	0.156
Hospital stay, mean ± SD (days)	3.57 ± 2.20	3.54 ± 1.03	3.84 ± 2.92	3.26 ± 1.78	0.491
Intraoperative complications, *n* (%)	1 (0.9)	0 (0)	0 (0)	1 (2.6)	0.390
Postoperative complications, *n* (%)	17 (15.6)	5 (19.2)	7 (15.6)	5 (13.2)	0.805
Type of postoperative complications, *n* (%)					0.145
No complication	92 (84.4)	21 (80.8)	38 (84.4)	33 (86.8)	
Hemorrhagic	2 (1.8)	0 (0)	2 (4.4)	0 (0)	
Infectious	4 (3.7)	1 (3.8)	3 (6.7)	0 (0)	
Urinary	0 (0)	0 (0)	0 (0)	0 (0)	
Lymphatic	0 (0)	0 (0)	0 (0)	0 (0)	
Nervous	2 (1.8)	1 (3.8)	0 (0)	1 (2.6)	
Thromboembolic	0 (0)	0 (0)	0 (0)	0 (0)	
Cardiovascular	5 (4.6)	1 (3.8)	2 (4.4)	2 (5.3)	
Suture dehiscence	2 (1.8)	0 (0)	0 (0)	2 (5.3)	
Others	2 (1.8)	2 (7.7)	0 (0)	0 (0)	
Clavien–Dindo classification, *n* (%)					0.756
I	3 (2.8)	1 (3.8)	2 (4.4)	0 (0)	
II	11 (10.1)	4 (15.4)	3 (6.7)	4 (10.5)	
IIIA	0 (0)	0 (0)	0 (0)	0 (0)	
IIIB	2 (1.8)	0 (0)	1 (2.2)	1 (2.6)	
IVA	0 (0)	0 (0)	0 (0)	0 (0)	
IVB	1 (0.9)	0 (0)	1 (2.2)	0 (0)	
V	0 (0)	0 (0)	0 (0)	0 (0)	

BMI: Body Mass Index EBL: Estimated Blood Loss. ICU: Intensive Care Unit.

**Table 3:** Nodal Mapping.

	Tot.	Group A (BMI > 40)	Group B (BMI 30–39)	Group C (BMI < 30)	*p* Value
Not attempted/ Failed nodal staging	4; 3.7%	1; 3.8%	2; 4.4%	1; 2.6%	0.907
Successful	93; 85.3%	19; 73.1%	43; 95.6%	31; 81.6%	0.026
Failed mapping and subsequent lymphadenectomy	12; 11.0%	6; 23.1%	0; -	6; 15.8%	0.006

BMI: Body Mass Index.

## Data Availability

The data used and materials to support the findings of this study are available from the corresponding author upon request.
